# Enzyme-Catalyzed Polymerization of Kraft Lignin from *Eucalyptus globulus*: Comparison of Bacterial and Fungal Laccases Efficacy

**DOI:** 10.3390/polym15030513

**Published:** 2023-01-18

**Authors:** Luisa García-Fuentevilla, Gabriela Domínguez, Raquel Martín-Sampedro, Manuel Hernández, María E. Arias, José I. Santos, David Ibarra, María E. Eugenio

**Affiliations:** 1 Forest Sciences Institute (ICIFOR-INIA), CSIC, Ctra. de la Coruña Km 7.5, 28040 Madrid, Spain; 2 Department of Biomedicine and Biotechnology, University of Alcalá, 28805 Alcalá de Henares, Spain; 3 General Services of Research SGIKER, University of the Basque Country (UPV/EHU), Edificio Joxe Mari Korta, Avda. Tolosa 72, 20018 San Sebastian, Spain

**Keywords:** bacterial laccase, central composite design, characterization, eucalypt, fungal laccase, kraft lignin, polymerization, response surface methodology

## Abstract

Kraft lignin, a side-stream from the pulp and paper industry, can be modified by laccases for the synthesis of high added-value products. This work aims to study different laccase sources, including a bacterial laccase from *Streptomyces ipomoeae* (SiLA) and a fungal laccase from *Myceliophthora thermophila* (MtL), for kraft lignin polymerization. To study the influence of some variables in these processes, a central composite design (CCD) with two continuous variables (enzyme concentration and reaction time) and three levels for each variable was used. The prediction of the behavior of the output variables (phenolic content and molecular weight of lignins) were modelled by means of response surface methodology (RSM). Moreover, characterization of lignins was performed by Fourier-transform infrared (FTIR) spectroscopy and different nuclear magnetic resonance (NMR) spectroscopy techniques. In addition, antioxidant activity was also analyzed. Results showed that lignin polymerization (referring to polymerization as lower phenolic content and higher molecular weight) occurred by the action of both laccases. The enzyme concentration was the most influential variable in the lignin polymerization reaction within the range studied for SiLA laccase, while the most influential variable for MtL laccase was the reaction time. FTIR and NMR characterization analysis corroborated lignin polymerization results obtained from the RSM.

## 1. Introduction

After cellulose, lignin is the second most abundant biopolymer on the planet [1]. It is synthetized from *p*-coumaryl alcohol (H), coniferyl alcohol (G), and sinapyl alcohol (S) monomers by enzymatic polymerization, in which oxidoreductase enzymes such as laccases and peroxidases are involved [2]. As a result, a heterogeneous complex tridimensional macromolecule is formed, containing different types of both ether (e.g., β-O-4′, α-O-4′, 5-O-4′, etc.) and carbon–carbon bonds (e.g., β-β′, β-5′, β-1′, 5-5′, etc.), and a wide variety of reactive groups depending on the biomass source. Accordingly, dicots (e.g., eucalypt, birch, poplar) contain around 20–25% of lignin, which is mostly composed of G and S units and traces of H units. On the other hand, gymnosperms (e.g., pine, spruce) with approximately 20–35% of lignin, are mostly composed of G units and very low proportions of H units. Finally, monocot grasses (e.g., flax, hemp, sisal) have lower lignin content (9–20%) composed of G and S units, together with high levels of H units [3]. Moreover, along with their source, the lignin isolation technology used strongly affects their features and properties and, therefore, the valorization ways [4].

Actually, the main source of lignin is the pulp and paper industry. Approximately 100 million tons per year of lignin were produced in 2015 with a value of roughly USD 732.7 million. Moreover, it is expected to increase to USD 913.1 million in 2025 [5]. Among the different pulp and paper processes, the kraft process is the most extended pulping technology, with an average lignin production estimated at 55–90 million tons per year [6]. Most of this kraft lignin is normally combusted, due to their high calorific value, to produce energy that is partially used in the same pulp and paper mills. However, this process generates an excess of energy, making the valorization of this waste lignin more interesting as high-added-value chemicals and materials that guarantee the sustainability and competitiveness of these mills [7]. In addition, kraft lignin valorization is also expected to benefit the future circular bioeconomy, which aims to maximize the usage and value of all raw materials, products, and wastes.

Although the inherent heterogeneity of kraft lignin (i.e., chemical composition, molecular structure, and molecular weight distribution) makes this waste material extraordinarily interesting, these features may be an obstacle for certain applications. To overcome this fact, the modification of the lignin structure is often a necessary step to produce the right lignin for each possible application [8]. The oxidative enzymes, such as laccases and peroxidases, involved in lignin biosynthesis in nature, can accomplish this modification [9]. Laccases (EC 1.10.3.2) are multicopper-containing oxidases with phenoloxidase activity, being widely expressed in nature, mainly in plants, insects, fungi, and bacteria [10]. The biological role of these enzymes is determined by their source and the phase of life of the organism producing them. For instance, fungal laccases participate in stress defense, morphogenesis, fungal plant–pathogen/host interactions, and lignin degradation, while bacterial laccases are involved in pigmentation, morphogenesis, toxin oxidation, and protection against oxidizing agents, and ultraviolet light [11]. These enzymes catalyze the oxidation of an extensive variety of phenolic and non-phenolic molecules, using oxygen as the final electron acceptor and releasing water as a by-product [11]. The catalytic site of laccases contains four copper ions. On the one hand, type-T1 copper, responsible for the characteristic blue color of the enzyme, is involved in the oxidation of the reducing substrate, acting as the primary electron acceptor. On the other hand, type-T2 copper together with two type-T3 coppers form a tri-nuclear copper cluster where oxygen is reduced to water [12]. The electrochemical potential of type-T1 copper is one of the most important properties of laccases, fluctuating between 0.4 and 0.8 V. Bacterial and plant laccases have low redox potential, whereas medium and high values are usually reported for fungal laccases [12].

The oxidative versatility, low catalytic requirements, and capacity of laccases to catalyze degradation or polymerization reactions make these enzymes suitable for a wide range of applications in different sectors, including lignocellulosic biorefinery, pulp and paper industry, food and textile sectors, bioremediation, and biosensor applications, among others [13]. More specifically, the enzymatic polymerization of lignin by laccases has been applied in the synthesis of new lignin-based polymeric materials [14] in, for example, the manufacture of green binders for fiberboard manufacturing [15], nanocomposite films formed by coating lignin nanoparticles along the microfibrilled cellulose fiber network [16], controlled-delivery fertilizer systems [17], and a pesticide release system [18]. In most of the studies on the enzymatic polymerization of lignin, fungal laccases are commonly used [15,17,18,19,20,21,22,23]. Only in recent years, bacterial laccases have also gained attention for this purpose [16,24,25]. Hence, there is a necessity to explore the potential of novel laccases, including bacterial enzymes, for kraft lignin polymerization.

This work aims to study the oxidative polymerization of *Eucalyptus globulus* kraft lignin by using different laccase sources, such as a bacterial laccase isolated from *Streptomyces ipomoeae* (SiLA) and a commercial fungal laccase from the ascomycete *Myceliophthora thermophila* (MtL). Laccase dosage and reaction time were the input variables evaluated at three levels to study laccase polymerization reactions, using a central composite design (CCD). The prediction of the behavior of the output variables (phenolic content and molecular weight of the resulting laccase-treated lignins) was modelled by means of response surface methodology (RSM). Moreover, structural characterization of the resulting lignins by Fourier-transform infrared spectroscopy (FTIR) and nuclear magnetic resonance (NMR) spectroscopy, as well as their antioxidant activity, were also evaluated.

## 2. Materials and Methods

### 2.1. Raw Material, Enzymes and Chemicals

Eucalypt (*Eucalyptus globulus*) residual lignin was isolated from kraft black liquor provided by La Montañanesa pulp mill (Lecta, Zaragoza, Spain). Then, the lignin was precipitated (pH of liquor lowered to 2.5 with concentrated sulfuric acid), centrifuged and washed with acid water (pH 2.5), dried and finally homogenized.

A recombinant bacterial laccase (SiLA) from *Streptomyces ipomoeae* CECT 3341.16 used for this study was overproduced and purified according to Guijarro et al. [26]. A commercial fungal laccase (MtL) from *Myceliophtora thermophila* (Novozym^®^ 51003) was also used, being kindly supplied by Novozymes (Bagsvaerd, Denmark). Enzyme activities were determined by oxidation of 5 mM 2,2′-azino-bis(3-ethylbenzothiazoline-6-sulphonic acid) (ABTS) to its cation radical (ε_436 nm_ = 29,300 M^−1^ cm^−1^) in 0.1 mM sodium acetate (pH 5) at 24 °C.

All the other reagents used were of analytical grade purchased either from Sigma–Aldrich (Madrid, Spain) or Merck (Barcelona, Spain).

### 2.2. Kraft Lignin Enzymatic Polymerization

Lignin was solubilized in phosphate buffer at pH 7.0 and 8.0 (100 mM), according to optimal pH for MtL and SiLA laccase activities, respectively [26,27], obtaining a solution of 1.5 g/L. The reactions took place at 60 °C and 45 °C, according also to optimal temperature for MtL and SiLA laccases, respectively [26,27]. At the end of reactions, the pH was lowered to 2.5 causing lignin precipitation. The reaction product was filtered and washed twice with acidified water (pH 2.5) and oven-dried at 40 °C under vacuum.

To study and optimize the influence of some variables on lignin polymerization, a central composite design (CCD) was used, considering two input variables (laccase dosage and reaction time) at three levels (−1, 0, +1). The design consisted of thirteen runs (experiments) from which five were center points and four were axial points (alpha = ±1.414). The values of laccase dosages (minimum value, 40 IU/g of kraft lignin; maximum value, 160 IU/g of kraft lignin) and reaction times (minimum value, 90 min; maximum value, 390 min) for the CCD were established based on previous experiments (Table 1) [28,29].

The output variables considered in this study were: the total phenolic content and the molecular weight of the resulting enzyme-treated lignins. Then, for each of the output variables, the obtained data from the CCD were used to fit a quadratic regression equation representing the influence of the two input variables. Each equation was plotted by means of response surface methodology (RSM). The design of the experiments of the CCD, the quadratic regression equations, and the plots obtained from modelling the equations by RSM were obtained using Minitab 19.1 software (Minitab Ltd., Coventry, United Kingdom).

### 2.3. Enzyme-Treated Lignins Characterization

#### 2.3.1. Total Phenolic Content

The total phenolic content of kraft lignins was determined following the Folin–Ciocalteu method with some modifications, according to Jiménez-López et al. [30]. Firstly, kraft lignin samples were dissolved in dimethylsulfoxide (DMSO). Then, 500 µL of Folin–Ciocalteau reagent was added to 100 µL of the sample dilution, followed by the addition of 400 µL of Na_2_CO_3_. The reaction mixture was incubated at 50 °C for 10 min, and absorbance at 760 nm were measured after cooling, using a UV–Vis spectrophotometer (Lambda 365, PerkinElmer, Boston, MA, USA). The total phenolic content of samples was quantified using a calibration curve prepared from a standard solution of gallic acid (1–200 mg/L) and expressed as mg gallic acid equivalent (GAE)/g of lignin (on a dry basis).

#### 2.3.2. Size Exclusion Chromatography (SEC)

SEC analysis (weight-average (Mw), number-average (Mn) molecular weights, and polydispersity (Mw/Mn)) of kraft lignins was carried out in an Agilent Technologies 1260 HPLC. The samples were analyzed at 254 nm (G1315D DAD detector, Agilent, Waldbronn, Germany) using two columns (Phenomenex) coupled in series (GPC P4000 and P5000, both 300 × 7.8 mm) and a safeguard column (35 × 7.8 mm). NaOH (0.05 M), pumped at a rate of 1 mL min^−1^, was employed as mobile phase at 25 °C for 30 min. Samples were dissolved at a final concentration of 0.5 g/L in NaOH (0.05 M). Polystyrene sulfonated standard (peak average molecular weights of 4210, 9740, 65,400, 470,000, PSS-Polymer Standards Service) were used for the calibration curve [31].

#### 2.3.3. Fourier-Transform Infrared (FTIR) and Ultraviolet–Visible (UV–Vis) Spectroscopy

FTIR spectra of kraft lignins were acquired by a JASCO FT/IR 460 Plus spectrometer (Jasco, Japan), equipped with an accessory single-reflection diamond, working with a resolution of 1 cm^−1^, 400 scans, and a spectral range of 600–2000 cm^−1^ [32].

UV–Vis analysis of kraft lignins, dissolved in 0.1 M NaOH to a final concentration of 50 µg mL^−1^, was carried out using a UV–Vis spectrophotometer (Lambda 365, PerkinElmer, Boston, MA, USA), and the absorbances were measured between λ 200 and 800 nm.

#### 2.3.4. Nuclear Magnetic Resonance Spectroscopy (NMR)

Solid-state ^13^C nuclear magnetic resonance (13C NMR) spectroscopy of kraft lignins was performed at 25 °C in a Bruker Avance III 400 MHz (Bruker, Billerica, MA, USA) at 100.64 MHz with the cross polarization/magic angle spinning (CP/MAS) technique. Lignin samples were prepared in 4 mm rotors and the spinning rate was 10 KHz. The contact time was 2 ms and the delay between scans was 5 s. The number of scans was 10,240 [32].

^13^C–^1^H two-dimensional nuclear magnetic resonance (2D NMR) spectra of kraft lignins were recorded at 25 °C in a Bruker AVANCE 500 MHz (Bruker, Billerica, MA, USA) equipped with a z-gradient double resonance probe. Around 40 mg of each lignin was dissolved in 0.75 mL of deuterated dimethylsulfoxide (DMSO-d6) and an HSQC (heteronuclear single quantum correlation) experiment was recorded. The spectral widths for the HSQC were 5000 Hz and 13,200 Hz for the ^1^H and ^13^C dimensions. The number of collected complex points was 2048 for ^1^H-dimension with a recycle delay of 5 s. The number of transients for the HSQC spectra was 64, and 256 time increments were always recorded in ^13^C-dimension. The J-coupling evolution delay was set to 3.2 ms. Squared cosine-bell apodization function was applied in both dimensions. Prior to Fourier transform the data matrices were zero filled up to 1024 points in the ^13^C-dimension. Residual DMSO (from DMSO-d6) was used as an internal reference (δ_C_/δ_H_ 39.6/2.5 ppm) [32].

#### 2.3.5. Antioxidant Activity of Kraft Enzyme-Treated Lignins

The antioxidant activity of kraft lignin samples was estimated following the ABTS^+•^ methods according to Re et al. [33] and Ratanasumarn et al. [34]. ABTS^+•^ was produced by the reaction between 2,2′-azino-bis(3-ethylbenzthiazoline-6-sulphonic acid) diammonium salt (ABTS) stock solution (7 mM) and potassium persulfate (2.45 mM). Prior to use, the ABTS^+•^ stock solution was diluted with ethanol to obtain an absorbance of 0.7 ± 0.02 at 734 nm. Then, 1 mL of the ABTS^+•^ stock solution was mixed with 10 μL sample (32 mg mL^−1^) or control and allowed to react for 6 min. The change in absorbance of the reaction mixture was measured at 734 nm and the antioxidant activity was expressed as trolox equivalent antioxidant capacity (TEAC). To calculate TEAC capacity, the gradient of the plot of the percentage inhibition of absorbance vs. concentration for lignin samples was divided by the gradient of the plot for trolox.

## 3. Results and Discussion

There is an increasing interest to evaluate the potential of novel laccases, including those from bacterial origin, for residual lignin polymerization. Presently, the fungal *M. thermophila* (MtL) laccase, with a high pH range and noticeable thermal stability, is widely employed for lignin polymerization [15,20,21,35]. *S. ipomoeae* (SiLA) laccase, with similar properties to MtL laccase, has been also assessed for this purpose [36], although to a lesser extent.

### 3.1. Effect of Laccase Dosage and Reaction Time on Total Phenolic Content

**Figure 1:**
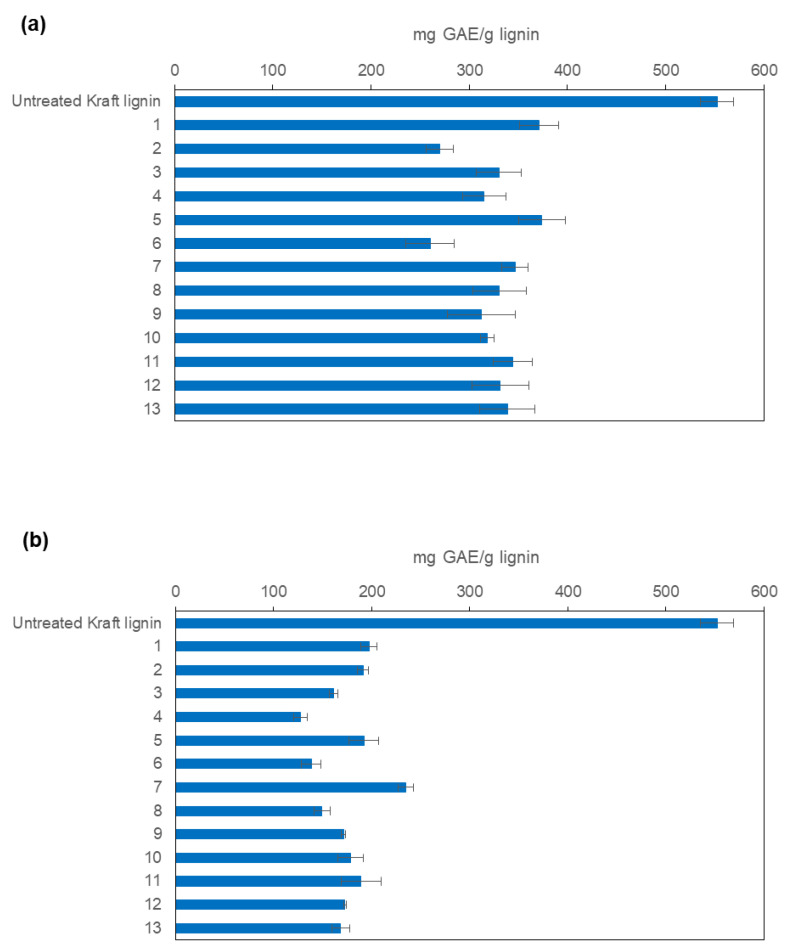
Total phenolic content of the resulting treated lignins with MtL (**a**) and SiLA (**b**) laccases expressed as mg GAE/g lignin (experiments resulting from CCD).

As it can be observed in Figure 1, both MtL and SiLA laccases showed the ability to reduce the phenolic content of the treated kraft lignins obtained from all experiments of the CCD. Nevertheless, the bacterial laccase achieved a higher reduction of total phenolic content compared to the fungal laccase, in spite of the similar low redox potential showed by both laccases (0.450 mV) [27,37].

The quadratic regression equation obtained from the phenolic content values (Figure 1a,b) when using MtL laccase (Equation (1)) and SiLA laccase (Equation (2)) over kraft lignin, and their regression coefficients are the following:

mg GAE/g lignin = 434.8 − 0.306 · Time (min) − 0.786 · Dosage (IU/g) − 0.000339 · Time (min) · Time (min) + 0.00089 · Dosage (IU/g) · Dosage (IU/g) + 0.002381 · Time (min) · Dosage (IU/g)

R-squared = 91.24%(1)

mg GAE/g lignin = 222.5 + 0.1458 · Time (min) − 0.588 · Dosage (IU/g) − 0.000343 · Time (min) · Time (min) + 0.00157 · Dosage (UA/g) · Dosage (IU/g) − 0.000776 · Time (min) · Dosage (IU/g)

R-squared = 92.93%(2)

**Figure 2:**
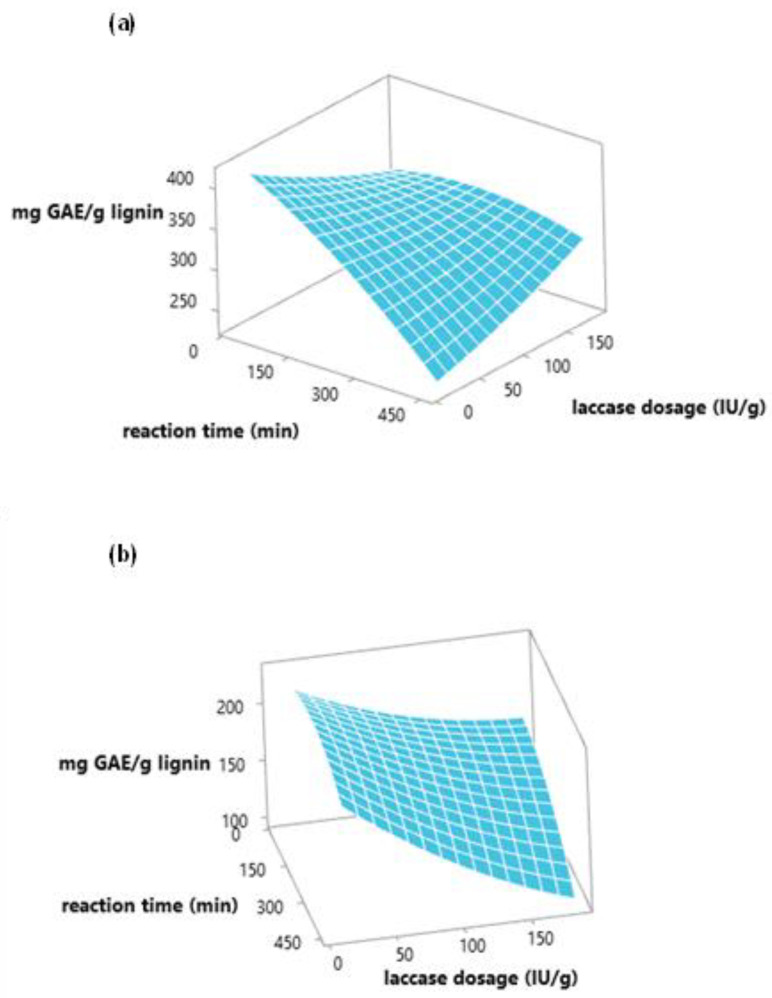
Response surface plot of total phenolic content expressed as mg GAE/g lignin to evaluate MtL (**a**) and SiLA (**b**) laccases dosage and reaction time.

Figure 2a,b show the response surface for lignin phenolic content as a function of enzyme concentration and reaction time using MtL or SiLA laccases, respectively. As it can be seen, the total phenolic content of lignin decreased with increasing reaction time in the studied region when MtL laccase was used. The maximum phenolic content reduction (53%) was obtained at 452 min using 100 IU/g of MtL laccase. In the case of SiLA laccase, the most influential variable on the total phenolic content reduction was the laccase dosage, observing a maximum decrease (77%) using 160 IU/g of SiLA laccase at 390 min.

As it is widely known, laccases can oxidize free phenolic lignin units, yielding resonance-stabilized phenoxyl radicals via a single electron transfer process [38]. Thus, the establishment of new linkages between the formed phenoxyl radicals leads to a lower content of free phenols in lignin. Different studies have already described the ability of MtL laccase to reduce the phenolic content of both kraft lignin and lignosulfonates. Then, the phenolic content was decreased by this fungal laccase by around 66% in the case of eucalypt kraft lignin [15], whereas a reduction of 52% was described using lignosulfonates [35]. On the other hand, the phenolic content reduction in lignin by bacterial laccases has been also reported. In this regard, Mayr et al. [25] showed the ability of CotA laccase to decrease the phenolic content between 30% and 65% in different kraft lignins of softwood and hardwood origin, respectively. Similar results were described by Wang et al. [16] when a commercial bacterial laccase (Metzyme^®^) was used to polymerize alkali lignins from birch and spruce materials.

Contrary to this study, the effects of laccase dosage and reaction time on the phenolic content of lignin are usually evaluated separately. Gillgren et al. [19] showed that longer reaction time resulted in higher reductions of phenolic content of both organosolv lignin and lignosulfonates when they were treated with a fungal laccase from *Trametes* (syn. *Coriolus polyporus*). A similar trend was reported by Huber et al. [35], using a laccase from MtL to polymerize both eucalypt kraft lignin and lignosulfonates. Moreover, these authors also observed a higher decrease in the phenolic content using higher MtL laccase dosages, indicating that the amount of enzyme used, together with reaction time, are important factors for the lignin polymerization process. Finally, Mayr et al. [25] also reported the influence of reaction time on the phenolic content decrease when a bacterial CotA laccase was used to polymerize different kraft lignins, observing a decrease in phenolic content by extending the reaction time.

### 3.2. Effect of Laccase Dosage and Reaction Time on Molecular Weight

**Figure 3:**
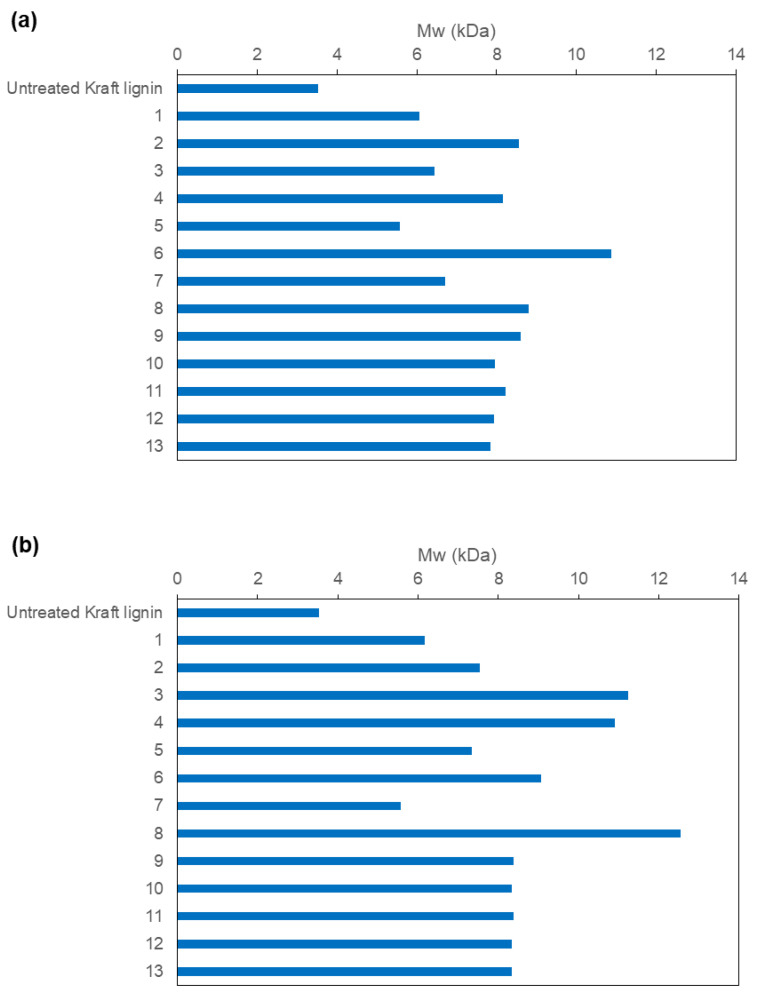
Weight-average (Mw) molecular weight values (KDa) of the resulting treated lignins with MtL (**a**) and SiLA (**b**) laccases (experiments resulting from CCD).

The molecular weight distributions of laccase-treated lignins are displayed in Figure S1. From them, weight-average (Mw) and number-average (Mn) molecular weights, as well as polydispersity (Mw/Mn) values were calculated (Table S1). In general, both MtL and SiLA laccases produced an increment in the Mw values of the treated kraft lignins obtained from all experiments (Figure 3a,b). Polydispersity values also showed higher values compared to the untreated lignin (Table S1).

The quadratic regression equation obtained from the Mw values (Figure 3a,b) when using MtL laccase (Equation (3)) and SiLA laccase (Equation (4)) over kraft lignin, and their regression coefficients are the following:

Mw (Da) = 3371 + 14.40 · Time (min) + 30.7· Dosage (IU/g) − 0.0050 · Time (min) · Time (min) − 0.0959 · Dosage (IU/g) · Dosage (UA/g) − 0.0223 · Time (min) · Dosage (IU/g)

R-squared = 83.75%(3)

Mw (Da) = 3936 + 7.42 · Time (min) + 25.46 · Dosage (IU/g) + 0.00040 · Time (min) · Time (min) + 0.1201· Dosage (IA/g) · Dosage (IU/g) − 0.0471 · Time (min) · Dosage (IU/g)

R-squared = 98.38%(4)

**Figure 4:**
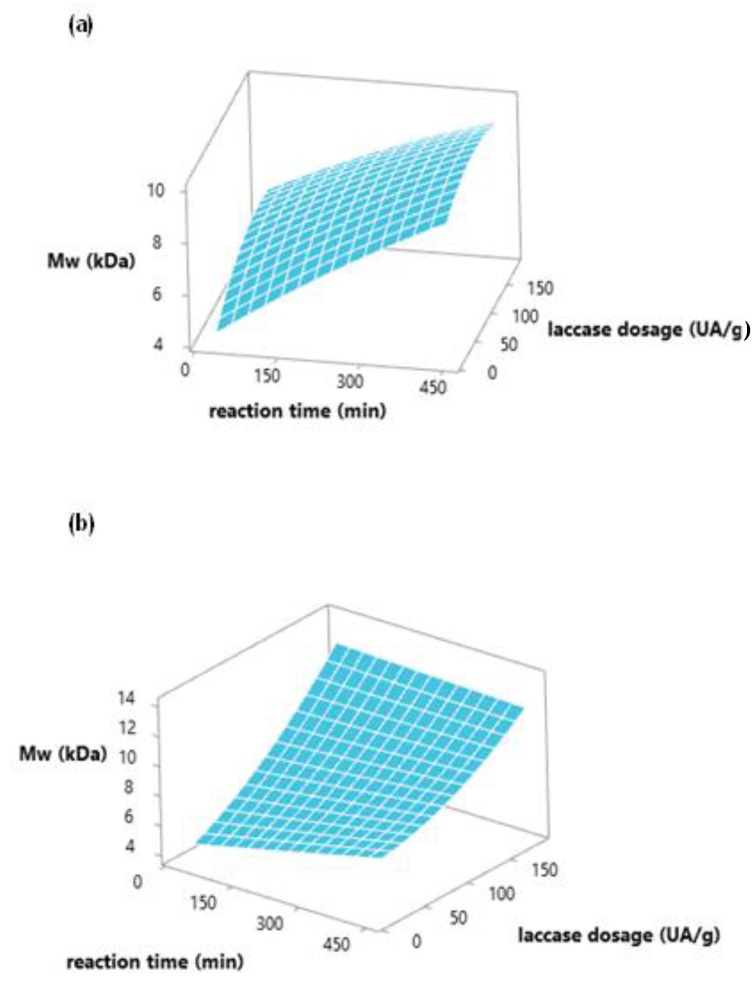
Response surface plot of weight-average (Mw) molecular weight values (KDa) to evaluate MtL (**a**) and SiLA (**b**) laccases dosage and reaction time.

Figure 4a,b show the response surface for lignin Mw as a function of enzyme concentration and reaction time of MtL and SiLA laccases, respectively. As can be observed, the molecular weight of the lignin increased by increasing the reaction time in the studied interval when MtL laccase was used, in agreement with the observed reduction in phenolic content (Section 3.1). The maximum molecular weight increment (3.0-fold, which correspond to the value of Mw 10,865 Da) was obtained at 452 min using 100 IU/g of MtL laccase. In the case of SiLA laccase, the most influential variable on the molecular weight was the laccase dosage used, as also observed for the phenolic content of lignin (Section 3.1). The maximum increment (3.5-fold, which corresponds to the value of Mw 12,545 Da) was obtained using 184.85 IU/g of SiLA laccase at 240 min.

As previously commented, the stabilized phenoxyl radicals, generated from lignin by laccase oxidation, undergo radical–radical coupling through phenyl ether–carbon and carbon–carbon linkages, yielding the observed increase in Mw values of kraft lignin by both laccases. Moreover, the polydispersity increase is also expected due to the non-selective radical–radical coupling reactions, which link lignin end groups to each other spontaneously with low or no control and, consequently leading to higher polydispersity values [20]. MtL laccase has already shown its capability to increase the molecular weight of both kraft lignin and lignosulfonates. Thus, Gouveia et al. [15] reported a strong increase (17.0-fold) in the average molecular weight of laccase-treated eucalypt kraft lignin (80,000 Da) compared to the untreated lignin sample (4700 Da). Huber et al. [35] also described a 12.0-fold increase in Mw (22,400 Da) for enzymatic polymerization of lignosulfonates (1900 Da for untreated lignin), and only a 1.4-fold increase (2300 Da) when kraft lignin (1600 Da for untreated lignin) was used. On the other hand, the molecular weight increase by bacterial laccases has been also described. Thus, Wang et al. [16] reported a 2.9-fold increase (from 17,750 Da for untreated lignin to 52,000 Da for laccase treated lignin) when a Metzyme^®^ laccase was used to polymerize alkali spruce lignin. Mayr et al. [25] achieved 6.0-fold increases in molecular weight for softwood (from 21,600 Da for untreated lignin to 130,000 Da for laccase-treated lignin) and 19.2-fold for hardwood kraft lignins (from 3150 Da for untreated lignin to 60,000 Da for laccase-treated lignin) when they were treated with a CotA laccase.

Similarly to phenolic content, the effects of laccase dosage and reaction time on the molecular weight of lignin are generally studied separately, reporting different results in function of both laccase and lignin sources. Gouveia et al. [21] observed that the major changes in molecular weight of kraft lignin treated with the fungal MtL laccase occurred during the first 2 h, although longer reaction time resulted in higher Mw values of the resulting treated lignins. These authors also showed a molecular weight increase as the enzyme dosage was augmented. In this regard, Areskogh et al. [39] determined that no significant increments in the molecular weight of lignosulfonates were observed at low MtL enzyme dosage, while the molecular weight increased by augmenting the enzyme concentration. Huber et al. [35] also demonstrated that the amount of biocatalyst used strongly influences the polymerization process. When 50 mg of MtL laccase was used, 4.0-fold and 1.7-fold molecular weight increments were determined for lignosulfonates and kraft lignin, respectively. However, when the MtL laccase was augmented to 100 mg, a 12.0-fold increase in the molecular weight was measured for enzymatic polymerization of lignosulfonates, and only a 1.4-fold increase was seen for kraft lignin. Finally, Mayr et al. [25] also achieved higher increases in the molecular weight of softwood and hardwood kraft lignins at longer reaction times using a bacterial CotA laccase.

While the significant phenolic content of kraft lignin can translate into good reactivity for producing phenol-formaldehyde resins, epoxy resins, polyester systems, and polyurethanes, among others, the molecular weight increase of kraft lignin by laccase enzymes enables new applications as lignin-based dispersants providing better adsorption properties, stabilizer for emulsions, and in thermoplastic blends or copolymers enhancing thermal and mechanical performance [32].

### 3.3. Antioxidant Activity

**Figure 5:**
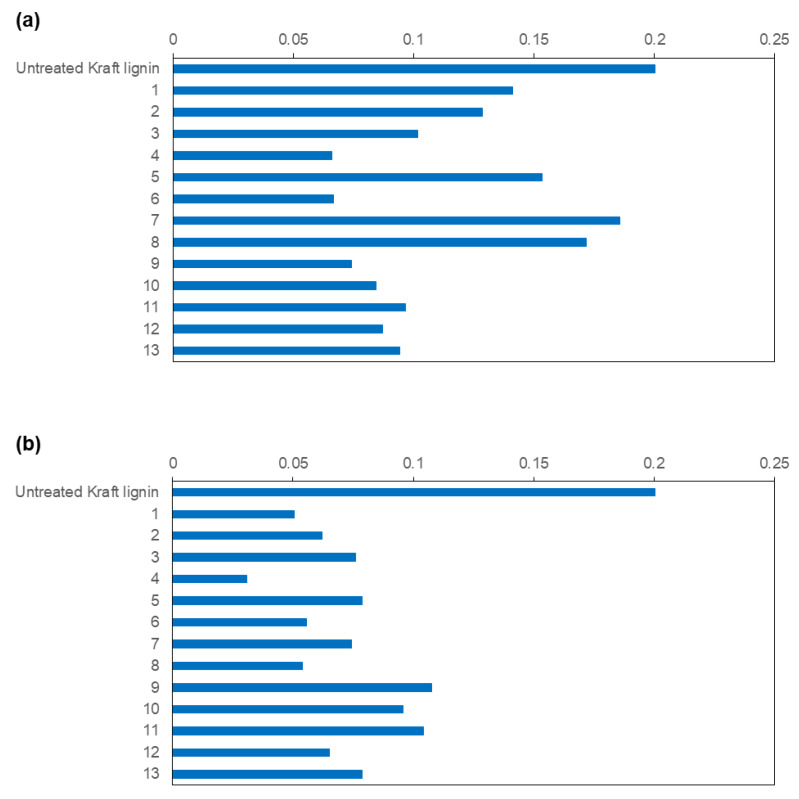
Antioxidant activity of the resulting treated lignins with MtL (**a**) and SiLA (**b**) laccases expressed as TEAC (experiments resulting from CCD).

The antioxidant ability of lignins (i.e., their capacity to act as radical scavengers) promotes their use as natural additives in food, cosmetics, pharmaceuticals, and polymeric formulations as an alternative to synthetic compounds such as butylated hydroxyanisole (BHA) and butylated hydroxytoluene (BHT), among others [40]. It is widely known that having a high phenolic content, low molecular weight, and narrow distribution seem to be favorable for the antioxidant capacity of lignin [40]. Nevertheless, in spite of the phenolic content decrease and molecular weight increase observed herein for the treated kraft lignins by both laccases, they still showed some antioxidant capacity, expressed as TEAC, (0.02–0.18) compared to the untreated sample (0.2) (Figure 5a,b for MtL and SilA laccases, respectively).

### 3.4. FTIR Characterization

**Figure 6:**
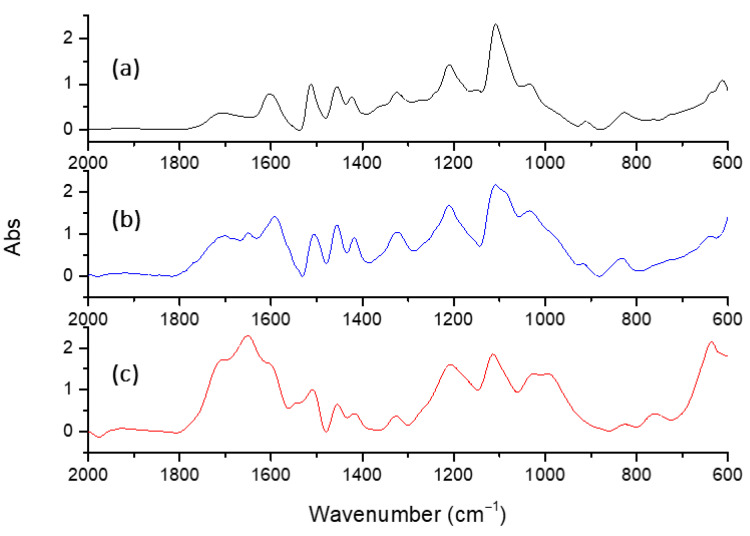
FTIR spectra, 2000–600 cm^−1^ region of the untreated lignin ((**a**), black line) and of the resulting treated lignins with MtL ((**b**), blue line) and SiLA ((**c**), red line) laccases. The bands in each spectrum are normalized with regard to the band at 1515 cm^−1^.

FTIR spectra of untreated and laccase-treated kraft lignins (MtL-KL and SiLA-KL resulting from experiments with maximum decrease in phenolic content and increase in molecular weight achieved) are displayed in Figure 6. The observed bands were assigned in comparison with others previously reported in the literature [41,42] and are displayed in Table S2. FTIR spectrum of kraft lignin showed the characteristic bands of lignin, which include those observed at 1610, 1515 and 1415 cm^−1^ associated with aromatic ring vibrations, and at 1455 cm^−1^ attributed to C−H asymmetric vibrations and deformations (Figure 6a). Bands attributed to syringyl (S) and guaiacyl (G) units were also identified, including those at 1315 cm^−1^ (S and G units), a shoulder at 1270 cm^−1^ (G units), 1220 cm^−1^ (G units), 1115 cm^−1^ (S units), 1025 cm^−1^ (G units) and 820 cm^−1^ (S units).

The major change in the FTIR spectra of MtL-KL and SiLA-KL samples compared to untreated kraft lignin spectra was observed at the bands corresponding to the C=O stretching for conjugated (1650 cm^−1^) and unconjugated (1715 cm^−1^) linkages (Figure 6b,c), as a consequence of the lignin oxidation caused by both laccases, being more noticeable in the case of the bacterial laccase. This effect was supported by UV–Vis, observing a decrease in the two absorption maxima at λ 230–240 and 280 nm, attributed to non-conjugated phenolic groups, in both MtL-KL and SiLA-KL samples due to lignin oxidation (Figure S2). Comparable results have been previously described by Gouveia et al. [15,21], when a laccase from *M. thermophila* was used for eucalypt kraft lignin polymerization, and Gillgren et al. [19], when a laccase from the white-fungus *C. polyporus* was employed to polymerize organosolv lignin and lignosulfonates. Moreover, both MtL-KL and SiLA-KL samples kept their characteristic triplet at 1610, 1515 and 1415 cm^−1^, which is indicative of no modification of lignin aromatic backbone, as previously observed by Areskogh et al. [43] during polymerization of lignosulfonates by *M. thermophila* laccase.

### 3.5. NMR Characterization

**Figure 7:**
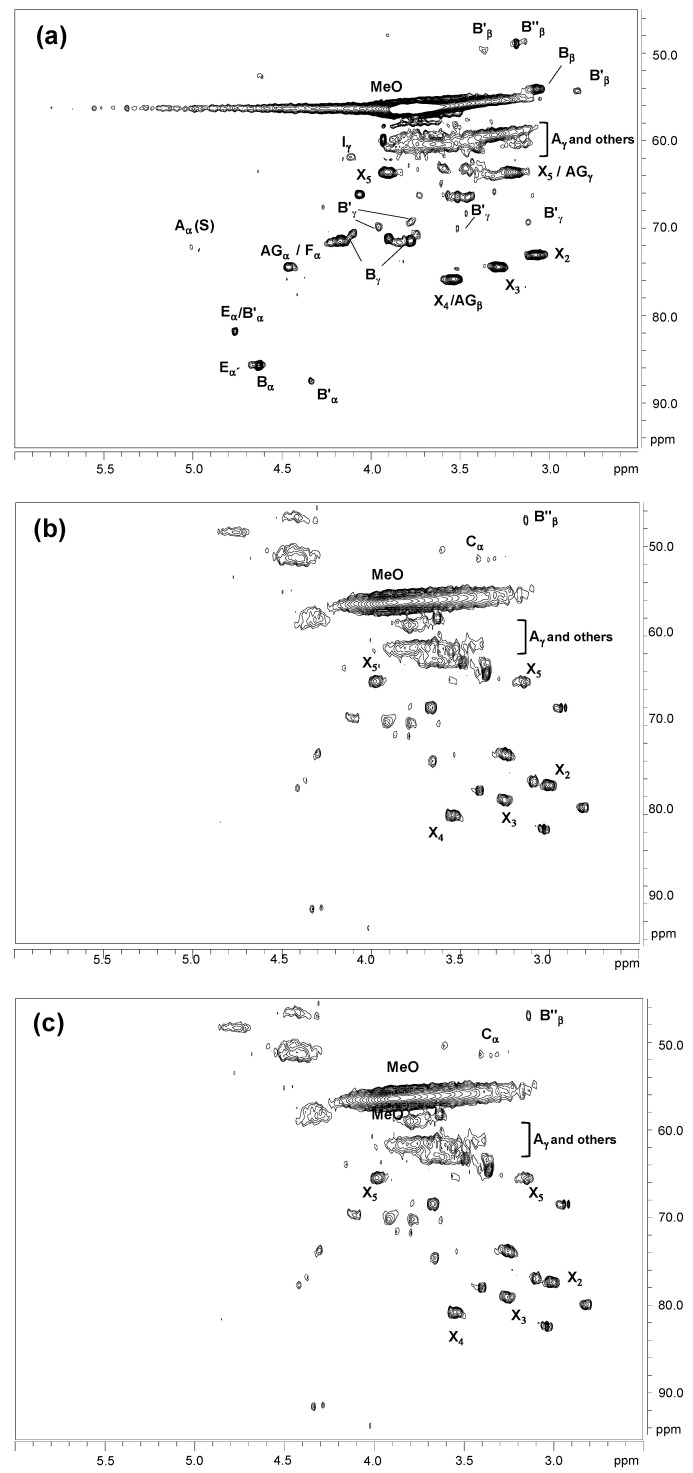
HSQC 2D-NMR spectra, δ_C_/δ_H_ 45.0–95.0/2.5–6.0 ppm aliphatic oxygenated region, of the untreated lignin (**a**) and of the resulting treated lignins with MtL (**b**) and SiLA (**c**) laccases. Correspondences between lignin structures and assigned letters in the figure: A, β-O-4′ alkyl-aryl ether; AG, aryl-glycerol; B, resinols; B′, epiresinols; B″, diaresinol; C, α-5′; E, spirodienones; F, Ar–CHOH–COOH; I, cinnamyl alcohol end-groups; X, xylopyranose (R, OH).

**Figure 8:**
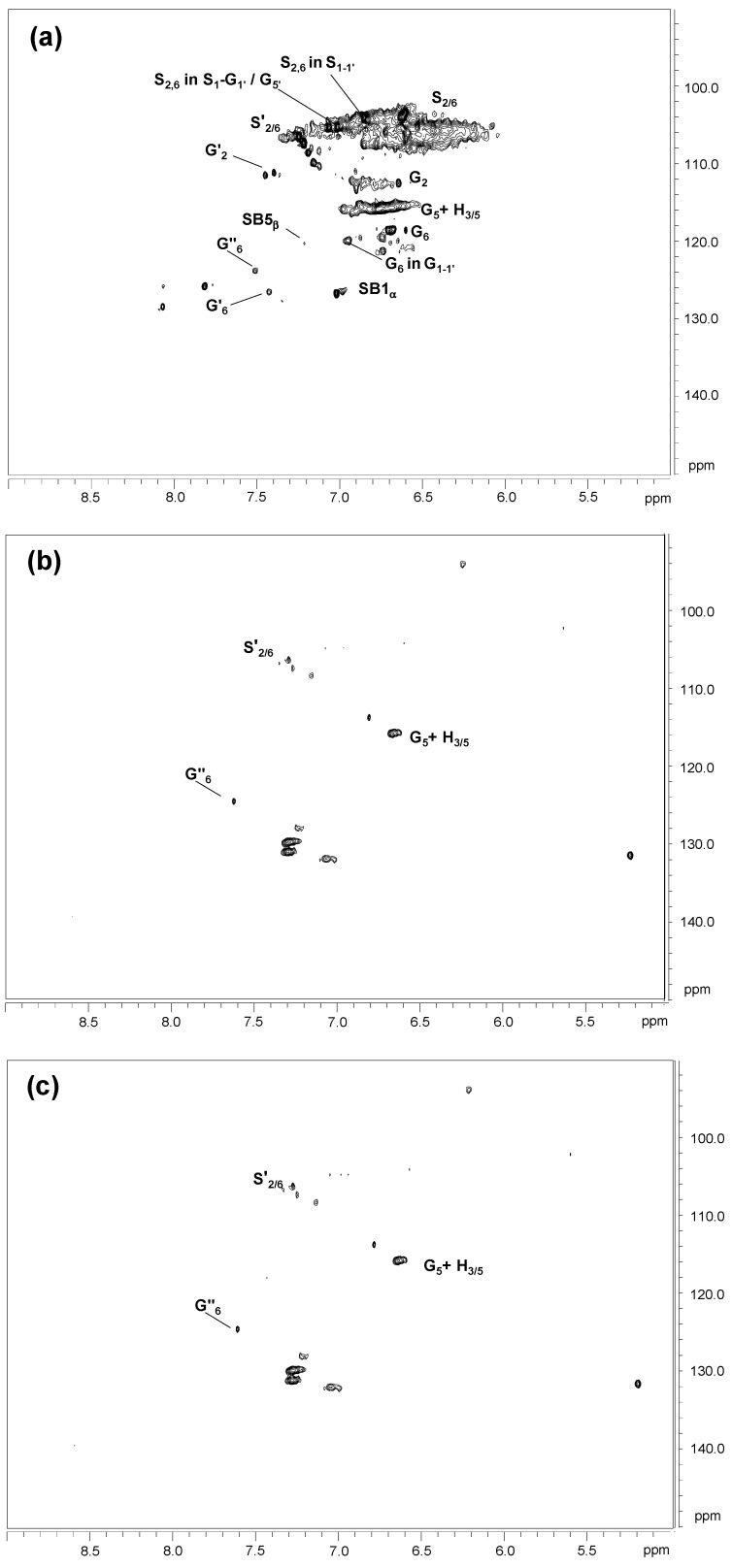
HSQC 2D-NMR spectra, δ_C_/δ_H_ 90.0–150.0/5.0–9.0 ppm aromatic region, of the untreated lignin (**a**) and of the resulting treated lignins with MtL (**b**) and SiLA (**c**) laccases. Correspondences between lignin structures and assigned letters in the figure: G, guaiacyl unit; G′, vanillin; G″, acetovanillona; H, *p*-hydroxyphenyl unit; S, syringyl unit; S′, syringaldehyde (R=H) or acetosyringone (R=CH_3_); S_1__–__1__′_, 3,5-tetramethoxy-para-diphenol; G_1__–__1__′_, 3-dimethoxy-para-diphenol; S_1_-G_1__′_/G_5__′_; SB1, stilbene-β-1′; SB5, stilbene-β-5′.

The HSQC spectra of untreated and laccase-treated kraft lignins (MtL-KL and SiLA-KL resulting from experiments with maximum decrease in phenolic content and increase in molecular weight achieved) are shown. They included the whole spectra (δ_C_/δ_H_ 0.0–150.0/0.0–9.0) in Supplementary Figure S3, and the spectra corresponding to the oxygenated aliphatic (δ_C_/δ_H_ 45.0–95.0/2.5–6.0 ppm) and the aromatic (δ_C_/δ_H_ 90.0–150.0/5.0–9.0 ppm) regions in Figure 7 and Figure 8, respectively. The main ^13^C–^1^H lignin correlation signals identified in HSQC spectra are displayed in Table S3, endorsed according to those described by the literature [42,44,45,46,47]. The lignin substructures identified are depicted in Figures S4 and S5.

The oxygenated aliphatic region of the kraft lignin spectrum exhibited information about the different interunit linkages present (Figure 7a), including those from native and kraft-derived linkages. Despite the well-known lignin degradation under alkaline conditions during kraft pulping [48], several remaining signals from native β-O-4′ and β-β′ resinol substructures were observed, as well as correlation signals for spirodienones and cinnamyl alcohol end-groups. Signals from kraft-derived lignin linkages could also be recognized. Among them, signals from epiresinols and diaresinol, both diastereomers from the transformation of the native resinol substructure during kraft pulping [47,49]. An aryl-glycerol substructure could also be hesitantly identified, produced from the non-phenolic β-aryl ether linkage under alkaline conditions during kraft pulping [50]. Finally, a correlation signal of lignin terminal structures with a carboxyl group in C_α_ (Ar–CHOH–COOH; F_α_), overlapping with aryl-glycerol, could also be found.

The aromatic region of kraft lignin spectra displayed the typical correlation signals of S, G, and H lignin units (Figure 8a), the usual pattern of hardwood lignins [51]. Moreover, a group of signals from lignin oxidation, such as oxidized S units, corresponding to syringaldehyde or acetosyringone, and oxidized G units, attributed to vanillin and acetovanillone, could also be detected. Signals from kraft-derived lignin linkages were also found in the aromatic region. Among them, correlation signals endorsed to β1 and β5 stilbene, derived from degradation of spirodienone and β-5′ phenylcoumaran during kraft pulping, respectively, were identified [46,47]. Finally, correlation signals from S_1-1′_ (3,5-tetramethoxy-para-diphenol), G_1-1′_ (3-dimethoxy-para-diphenol) and S_1_-G_1′_/G_5′_ were tentatively identified as a result of C_α_-C_1_ breakdown in a retro-aldol reaction, followed by a radical coupling reaction, during kraft pulping [32,45,46].

Significant changes in the oxygenated aliphatic region of MtL-KL and SiLA-KL spectra (Figure 7b,c for MtL-KL and SiLA-KL samples, respectively), compared to the kraft lignin spectrum (Figure 7a), were found. In general, a complete disappearance of signals assigned to native and kraft-derived linkages was observed for both MtL-KL and SiLA-KL lignin samples, probably due to the cleavage of interunit linkages by treatment with both laccases. Nevertheless, some signals from β-O-4′ and diaresinol were still found. In this sense, Prasetyo et al. [23] reported a decrease in the intensity signals of β-O-4′ linkages when lignosulfonates were treated with *Trametes villosa* and *Trametes hirsuta* laccases. Wang et al. [16] also described a cleaved of β-aryl ether and β-β′ resinol substructures during the treatment of alkali lignins with a commercial (MetZyme^®^) bacterial laccase. In addition to the cleavage of interunit linkages, a new signal could also be observed in the aliphatic oxygenated region of both MtL-KL and SiLA-KL spectra, which was tentatively attributed to α-5′ condensed structures. The appearance of this structure is probably due to lignin condensation/polymerization reactions by laccases action. In this sense, Wang et al. [16] already described the formation of this condensed structure during the treatment of alkali lignins with the MetZyme bacterial laccase.

MtL-KL and SiLA-KL spectra also showed a near complete disappearance of the aromatic ^13^C–^1^H correlation signals (Figure 8b,c) compared to the kraft lignin spectrum (Figure 8a). Nevertheless, some intensity of signals corresponding to G and H units as well as to lignin oxidation, such as oxidized S and G units, could still be found. This important loss of aromatic correlation signals observed in the HSQC spectra after the enzymatic treatment with both laccases could indicate a significant modification of the lignin aromatic backbone. However, when laccase-treated lignins were analyzed by 1D-NMR, it could be inferred that the loss of aromatic correlation signals observed by 2D-NMR was due to deprotonation of the lignin benzene rings, as revealed by the ^1^H NMR MtL-KL and SiLA-KL spectra (Figure S6). Meanwhile strong signals of aromatic carbons could be seen in the ^13^C-NMR MtL-KL and SiLA-KL spectra (Figure S7), proving that benzene rings were not degraded by laccase treatment, as previously seen by FTIR analysis (Section 3.4). The decrease or complete disappearance of aromatic proton signals was not entirely unexpected, as it has been previously described in lignosulfonates by the action of *T. villosa* and *T. hirsuta* laccases [23], in eucalypt kraft lignin treated with *M. thermophila* laccase [15], in alkali lignins by the action of MetZyme bacterial laccase [16], and in organosolv lignin and lignosulfonates treated with *C. polyporus* laccase [19]. All these authors have related this effect with the formation of condensed structures such as 5-5′ or 4-O-5′.

^13^C-NMR of laccase treated lignins (Figure S7b,c) also showed a remarkable increase in the signal at δ_C_ 176 ppm (carbonyl groups), especially in the SiLA lignin sample as previously described by FTIR analysis (Section 3.4), resulting from lignin oxidation caused by laccases action. A significant decrease in the signals at δc 147 ppm, corresponding to C_3_ and C_5_ of phenolic S units and C_3_ and C_5_ in phenolic G units, and at δc 134 ppm, endorsed to C_1_ and C_4_ in phenolic S units and C_1_ in phenolic G units, was also observed, resulting from the phenolic lignin units’ oxidation by laccase treatment, which supports the phenolic content decrease observed in Section 3.1. At a time, an increase in the shoulders at δc 152 ppm and at δc 130 ppm, from non-phenolic lignin units were also visible. Santos et al. [52] assigned the signal at δc 152 ppm to C_3_ in new 5-5′ or C_3_ (and C_4_/C_5_) in new 4-O-5′ structures formed during laccase (*T. villosa*) treatment of lignosulfonates, whereas Magina et al. [53] endorsed the signal at δc 130 ppm to C_5_ in 5-5′ structure during MtL laccase treatment of lignosulfonates.

**Figure 9:**
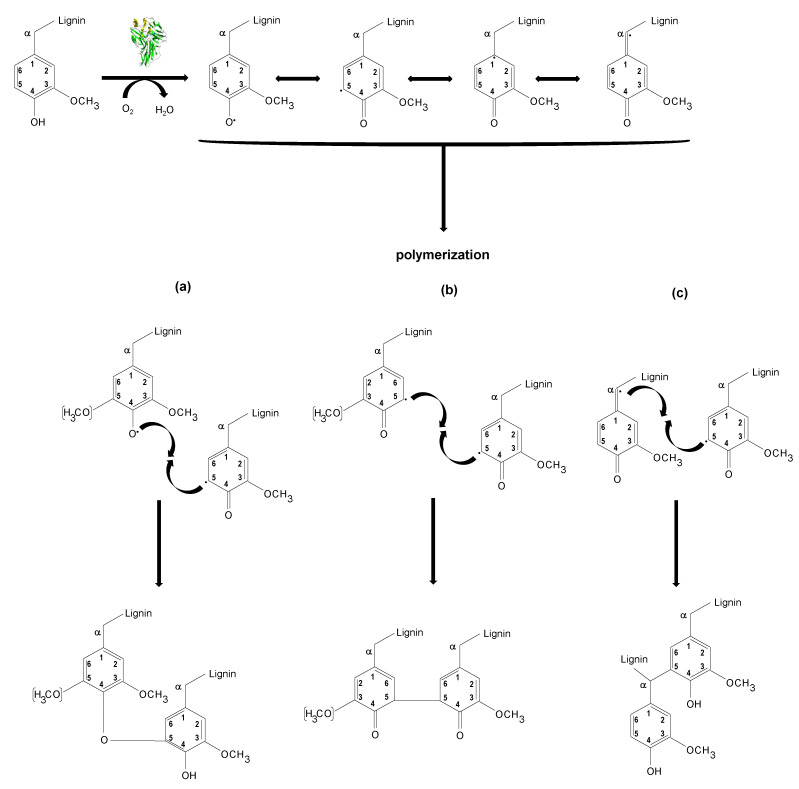
Schematic representation of oxidative lignin polymerization catalyzed by laccase. Radical coupling leads to formation of new condensed structures such as 4-O-5′ (**a**), 5-5′ (**b**), and α-5′ (**c**).

Then, these observations arising from NMR analysis suggest lignin condensation/polymerization reactions by laccases action, supporting the molecular weight increment observed by SEC (Section 3.2). The phenoxy radicals formed by the action of the laccase enzymes on the phenolic units present in the initial kraft lignin together with those derived from the cleavage of interunit linkages underwent radical–radical coupling through phenyl ether–carbon and carbon–carbon links resulting in new condensed structures such as α-5′, 5-5′, and 4-O-5′ (Figure 9).

## 4. Conclusions

In order to produce the most appropriate lignin for each possible application, structural modification of this molecule is needed. A possible way to achieve this goal is by using laccase enzymes, which is considered a sustainable and environmentally friendly approach. Concerning this, this study confirmed the ability of a bacterial laccase from *S. ipomoeae* (SiLA) and a commercial fungal laccase from *M. thermophila* (MtL) to polymerize kraft lignin (referring to polymerization as lower phenolic content and higher molecular weight). Specifically, the enzyme dosage was the most influential variable in the kraft lignin polymerization reaction, within the range studied, when the bacterial SiLA laccase was used, while for MtL fungal laccase the most influential variable was the reaction time. FTIR and NMR characterization spectra verified lignin polymerization, observing new condensed structures such as α-5′, 5-5′, and 4-O-5′.

## Figures and Tables

**Table 1:** Experimental conditions of the central composite design for investigation of kraft lignin polymerization by SiLA and MtL laccases considering two input variables (laccase dosage and reaction time) at three levels (−1, 0, +1).

	Coded Levels		Experimental Values	
Run	Reaction Time	Laccase Dosage	Reaction Time (Min)	Laccase Dosage (IU/g)
1	−1	−1	90.0	40.0
2	1	−1	390.0	40.0
3	−1	1	90.0	160.0
4	1	1	390.0	160.0
5	−1.414	0	27.87	100.0
6	1.414	0	452.13	100.0
7	0	−1.414	240.0	15.15
8	0	1.414	240.0	184.85
9	0	0	240.0	100.0
10	0	0	240.0	100.0
11	0	0	240.0	100.0
12	0	0	240.0	100.0
13	0	0	240.0	100.0

## Data Availability

Not applicable.

## Supplementary Materials

The following supporting information can be downloaded at: https://www.mdpi.com/article/10.3390/polym15030513/s1, Figure S1. Molecular weight distributions of the resulting treated lignins with MtL (a) and SiLA (b) laccases. Figure S2. UV–Vis spectra, λ 200–800 nm, of the untreated lignin (a) and of the resulting treated lignins with MtL (b) and SiLA (c) laccases. Figure S3. HSQC 2D-NMR whole spectra, δ_C_/δ_H_ 45.0–95.0/2.5–6.0 ppm, of the untreated lignin (a) and of the resulting treated lignins with MtL (b) and SiLA (c) laccases. Figure S4. Main lignin and carbohydrate substructures identified in aliphatic oxygenated region of the untreated kraft lignin and of the resulting treated lignins with MtL and SiLA laccases. Figure S5. Main lignin substructures identified in aromatic region of the untreated kraft lignin and of the resulting treated lignins with MtL and SiLA laccases. Figure S6. ^1^H NMR spectra, δ_H_ 0.0–9.0 ppm, of the untreated kraft lignin (a) and of the resulting treated lignins with MtL (b) and SiLA (c) laccases. Figure S7. ^13^C NMR spectra, δ_C_ 0.0–200.0 ppm, of the untreated kraft lignin (a) and of the resulting treated lignins with MtL (b) and SiLA (c) laccases. Table S1. Weight-average (Mw) and number-average (Mn) molecular weights and polidispersity (Mw/Mn) of the untreated kraft lignin and of the resulting treated lignins with MtL and SilA laccases. Mw and Mn are given in Da. Table S2. Main assignments of untreated and laccase-treated kraft lignins FTIR bands. Table S3. Assignment of main lignin and carbohydrates ^13^C–^1^H correlation signals in the HSQC spectra of untreated kraft lignin and the resulting treated lignins with MtL and SiLA laccases.
