# Supplementary material for: Enzyme-Catalyzed Polymerization of Kraft Lignin from Eucalyptus globulus: Comparison of Bacterial and Fungal Laccases Efficacy
Source: Polymers (Basel). 2023 Jan 18;15(3):513. doi: 10.3390/polym15030513 (PMC9920240; doi:10.3390/polym15030513)
Supplement: Supplementary file 1 [file polymers-15-00513-s001.zip › polymers-2141192-supplementary.pdf]

a

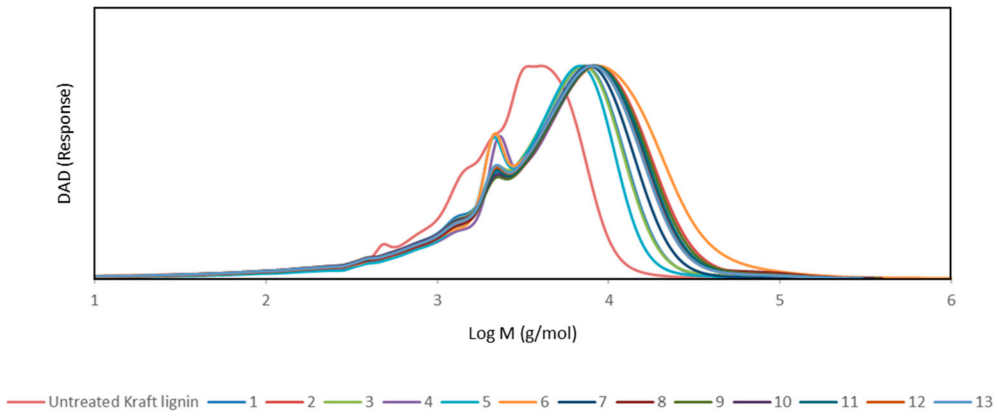

b

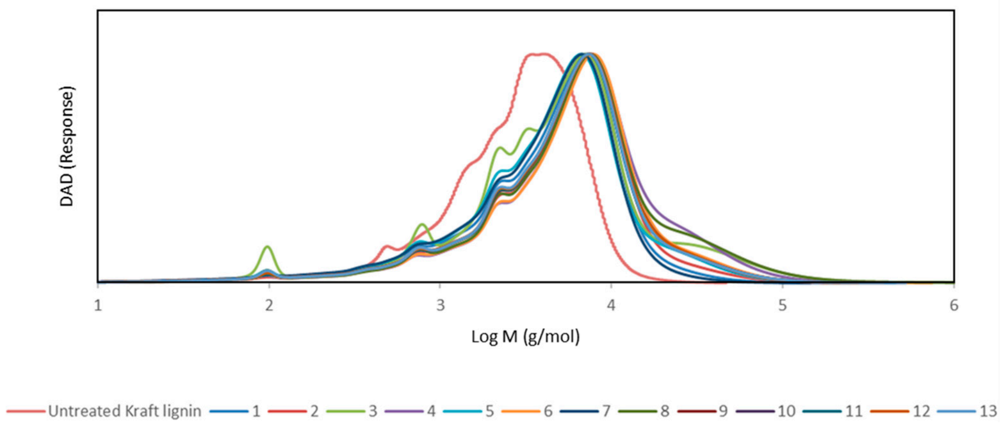

Figure S1. Molecular weight distributions of the resulting treated lignins with MtL (**a**) and SiLA (**b**) laccases.

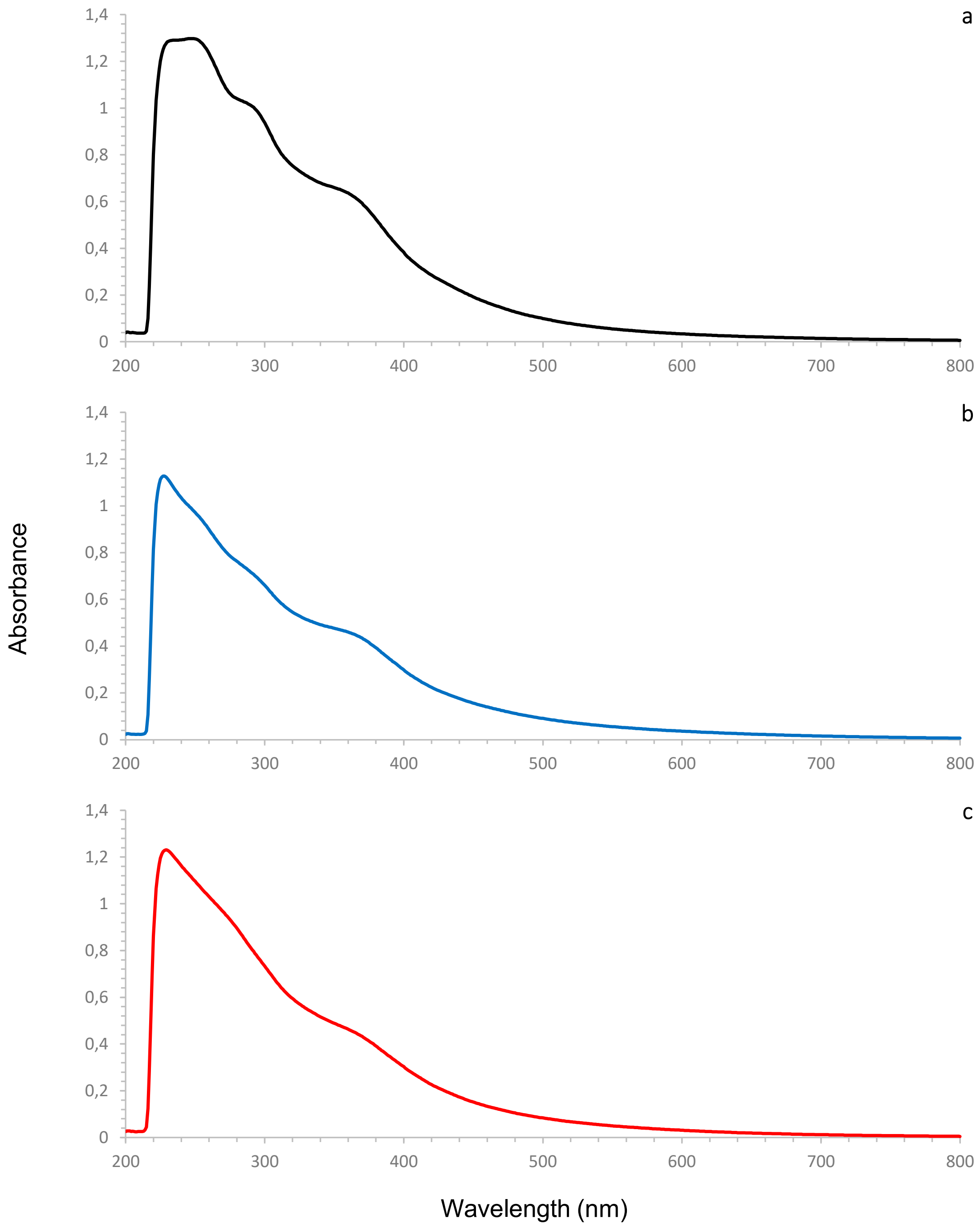

Figure S2. UV-Vis spectra, λ 200-800 nm, of the untreated lignin (**a**) and of the resulting treated lignins with MtL (**b**) and SiLA (**c**) laccases.

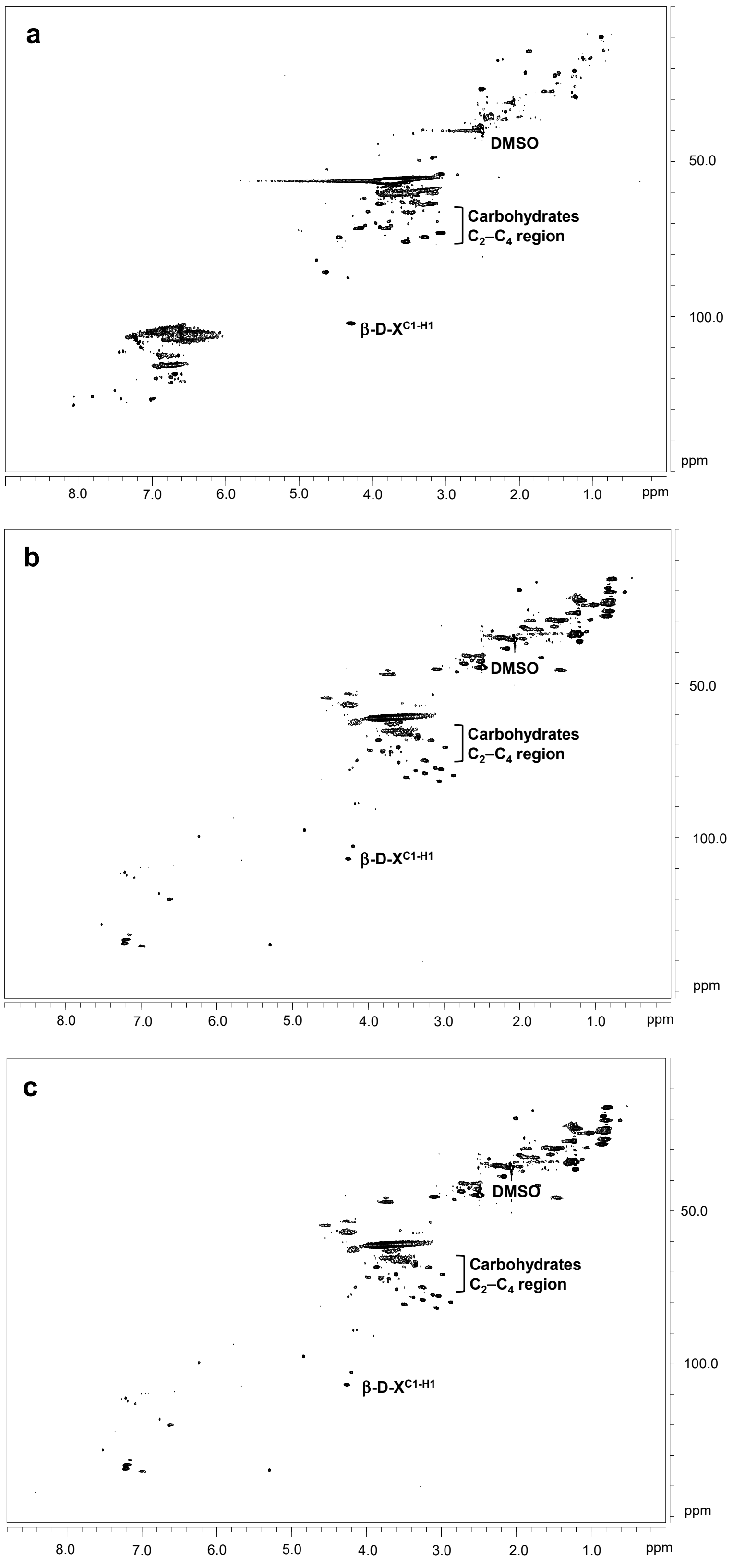

Figure S3. HSQC 2D-NMR whole spectra, δC/δH 0.0–150.0/0.0–9.0 ppm, of the untreated lignin (**a**) and of the resulting treated lignins with MtL (**b**) and SiLA (**c**) laccases.

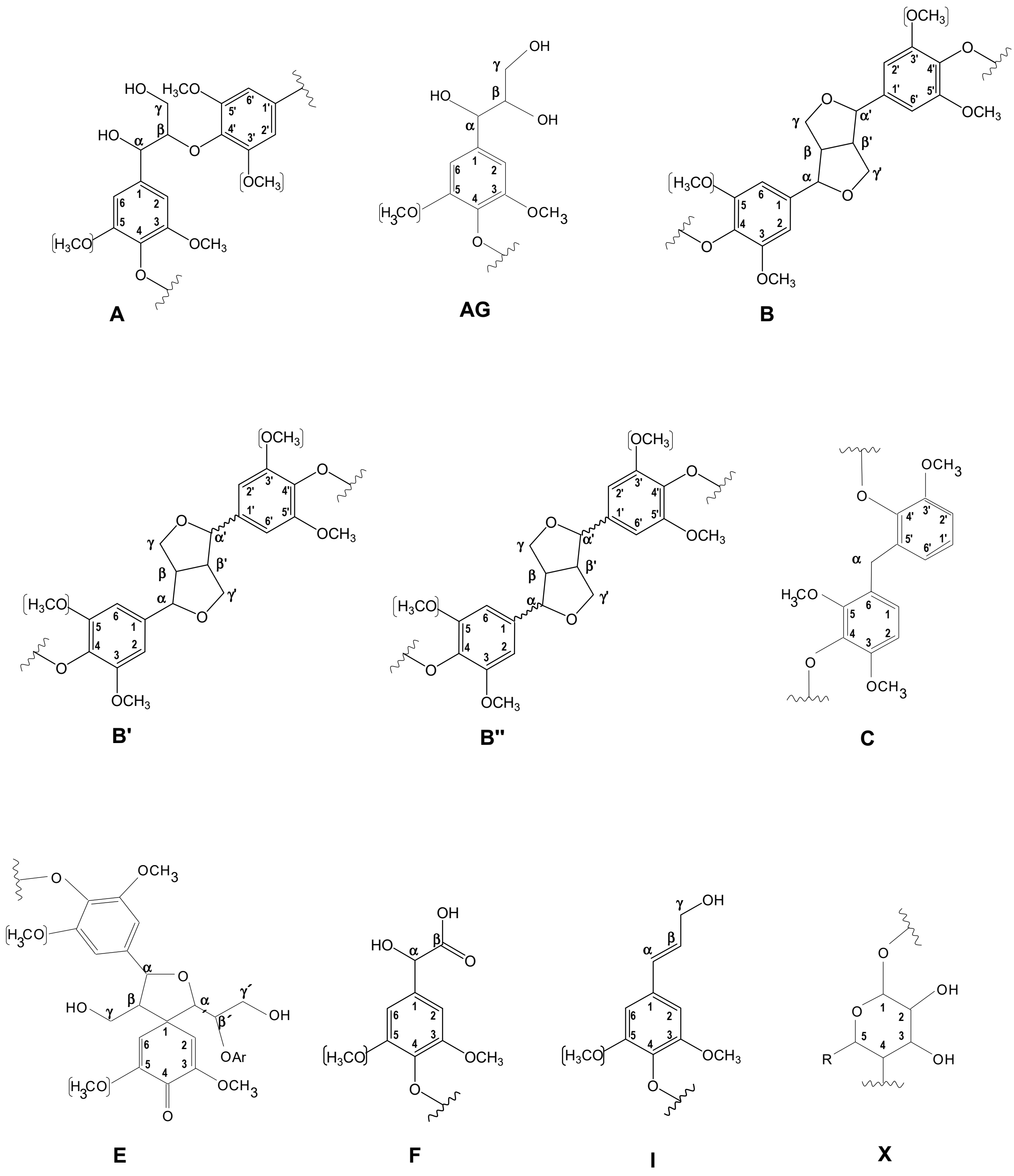

Figure S4. Main lignin and carbohydrate substructures identified in aliphatic oxygenated region of the untreated Kraft lignin and of the resulting treated lignins with MtL and SiLA laccases.

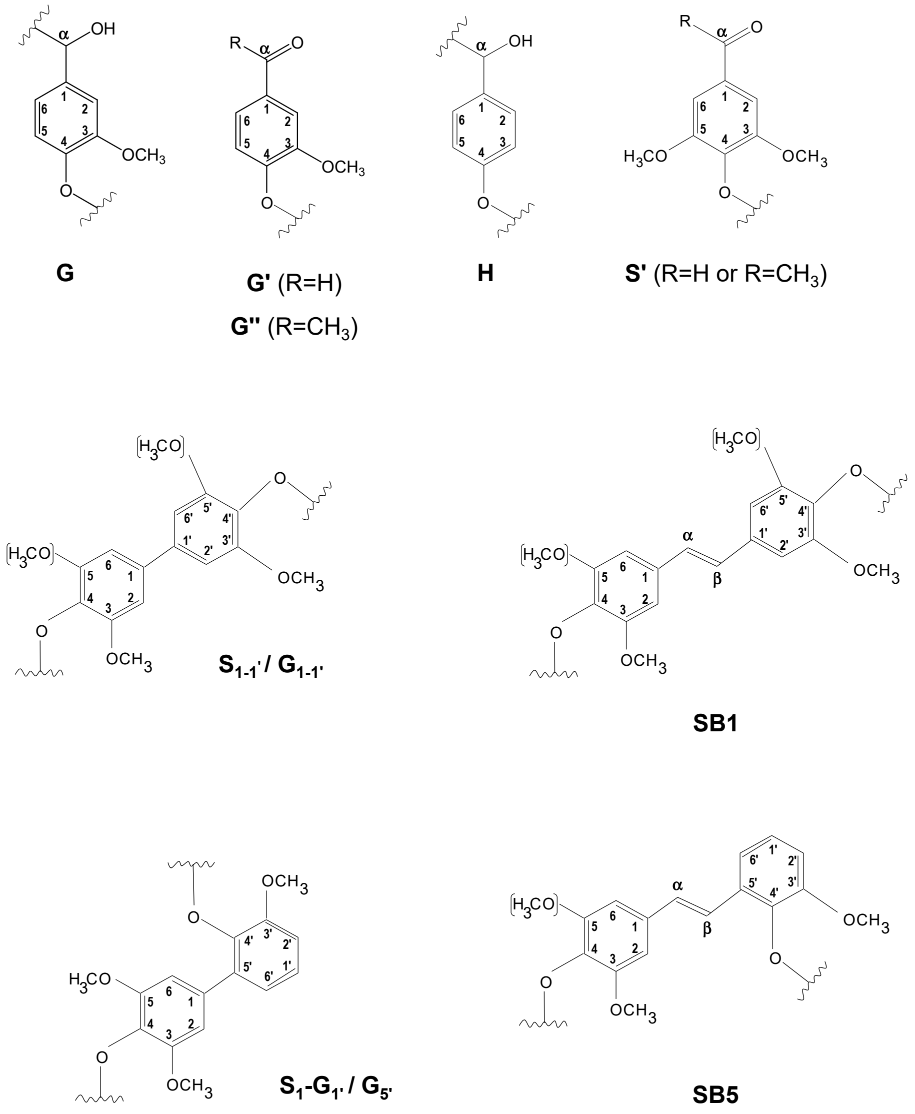

Figure S5. Main lignin substructures identified in aromatic region of the untreated Kraft lignin and of the resulting treated lignins with MtL and SiLA laccases.

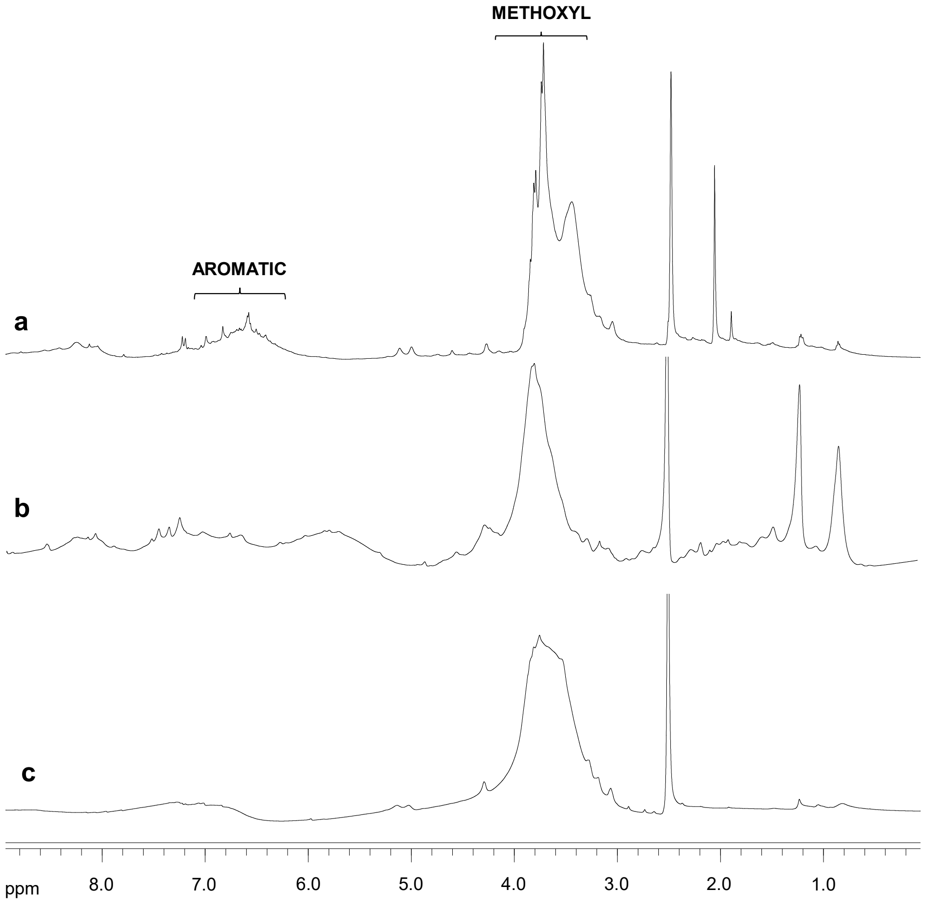

Figure S6. ^{1}H NMR spectra, δ_H 0.0–9.0 ppm, of the untreated Kraft lignin (**a**) and of the resulting treated lignins with MtL (**b**) and SiLA (**c**) laccases.

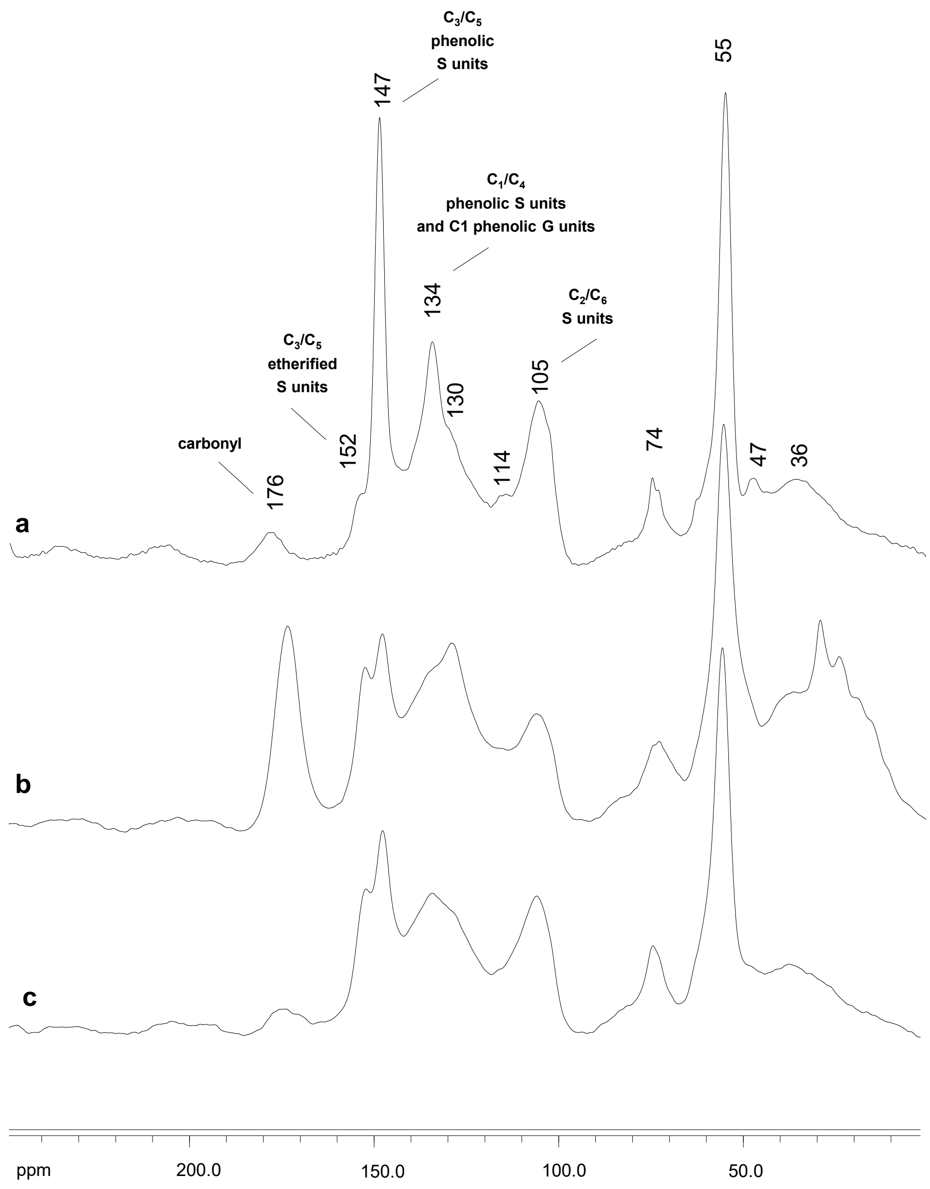

Figure S7. ^{13}C NMR spectra, δ_C 0.0–250.0 ppm, of the untreated Kraft lignin (**a**) and of the resulting treated lignins with MtL (**b**) and SiLA (**c**) laccases.

Table S1. Weight average (Mw) and number-average (Mn) molecular weights and polidispersity (Mw/Mn) of the untreated Kraft lignin and of the resulting treated lignins with MtL and SilA laccases. Mw and Mn are given in Da.

	**Sample**	**Mw**	**Mn**	**D**
Starting material		3530.4	526.99	6.6993
Lignin treated with MtL	1	6054	517.58	11.697
	2	8566.7	474.62	18.022
	3	6452	548.4	11.765
	4	8162.7	330.04	24.732
	5	5580	668.5	8.3471
	6	10865	394.97	27.509
	7	6715.6	540.2	12.431
	8	8802.5	665.11	13.235
	9	8599.8	494.04	17.407
	10	7963.6	530.15	15.021
	11	8220.4	597.91	13.749
	12	7923.4	620.81	12.763
	13	7836.5	435.39	17.999
Lignin treated with SiLA	1	6177.9	701.67	8.8047
	2	7537.4	849.88	8.8689
	3	11248	667.12	16.861
	4	10911	846	12.897
	5	7335.3	792.61	9.2547
	6	9074.1	746.65	12.153
	7	5558.1	557.57	9.9683
	8	12545	790.44	15.871
	9	8391.3	710.59	11.809
	10	8333.8	682.95	12.203
	11	8373.1	714.31	11.722
	12	8346	708.62	11.778
	13	8339.9	720.63	11.573

Table S2. Main assignments of untreated and laccase-treated Kraft lignins FTIR bands

Wavenumber	Characteristic groups
1715 cm^{-1}	C=O stretching for unconjugated linkages
1650 cm^{-1}	C=O stretching for conjugated linkages
1610 cm^{-1}	Vibrations of the aromatic ring
1515 cm^{-1}	Vibrations of the aromatic ring
1455 cm^{-1}	C−H asymmetric vibrations and deformations
1415 cm^{-1}	Vibrations of the aromatic ring
1315 cm^{-1}	Aromatic ring breathing (S and G condensed units)
1270 cm^{-1}	Aromatic ring breathing with C=O stretching (G units)

1220 cm^{-1}	Aromatic ring breathing with C–C, C–O, and C=O stretching (G units)
1115 cm^{-1}	C–H bond deformation (S units)
1025 cm^{-1}	C–H bond deformation (G units)
820 cm^{-1}	C–H out of plane deformation (S units)

Table S3. Assignment of main lignin and carbohydrates ^{13}C-^{1}H correlation signals in the HSQC spectra of the untreated Kraft lignin and of resulting treated lignins with MtL and SiLA laccases.

δ_C/δ_H (ppm)	Assignment
48.8/3.19	C_β–H_β, diaresinol substructures (**B′′**)
49.7/3.35	C_β–H_β, epiresinol substructures (**B′**)
51.9/3.4	C_α–H_α, α-5′ condensed substructure (**C**)
53.8/3.05	C_β–H_β, resinol substructures (**B**)
54.0/2.82	C_β–H_β, epiresinol substructures (**B′**)
56.0/3.71	C–H, methoxyls (**MeO**)
60.6/3.40–3.64	C_γ–H_γ, **β**-O-4′ substructures (**A**)
61.8/4.12	C_γ–H_γ, cinnamyl alcohol end groups (**I**)
63.4/3.23–3.88	C_5–H_5, xylan
63.6/3.10	C_γ–H_γ, aryl-glycerol (**AG**)
69.3/3.30–3.70	C_γ–H_γ, epiresinol substructures (**B′**)
70.1/3.73–4.10	C_γ–H_γ, epiresinol substructures (**B′**)
71.3/3.77–4.16	C_γ–H_γ, resinol substructures (**B**)
72.3/4.87	C_α–H_α, **β**-O-4′ **S** unit (**A**)
73.0/3.08	C_2–H_2, xylan
74.0/4.41	C_α–H_α, aryl-glycerol (**AG**)
74.3/3.31	C_3–H_3, xylan
74.3/4.43	C_α–H_α, Ar–CHOH–COOH units (**F**)
75.6/3.47	C_β–H_β aryl-glycerol (**AG**)
75.9/3.52	C_4–H_4, xylan
81.6/4.75	C_α–H_α, spirodienone substructures (**E**)
81.8/4.76	C_α–H_α, epiresinol substructures (**B′**)
85.5/4.76	$C_{\alpha'}$–$H_{\alpha'}$, spirodienone substructures (**E**)
85.3/4.63	C_α–H_α, resinol substructures (**B**)
87.5/4.30	C_α–H_α, epiresinol substructures (**B′**)
101.9/4.30	C-1, (1-4) **β**-D-Xylp
104.1/6.61	$C_{2,6}$–$H_{2,6}$, S units (**S**)
103.9/6.83	$C_{2,6}$–$H_{2,6}$, 3,5-tetramethoxy-*para*-diphenol substructures ($\mathbf{S}_{1\text{-}1'}$)
105.0/6.9	$C_{2,6}$–$H_{2,6}$, $\mathbf{S}_1$-$\mathbf{G}_{1'}$ / $\mathbf{G}_{5'}$ substructures
107.0/7.30	$C_{2,6}$–$H_{2,6}$, oxidized (H–C_α=O or H_3C–C_α=O) S units (**S′**)
110.8/6.90	C_2–H_2, G units (**G**)
111.3/7.38	C_2–H_2, oxidized (H–C_α=O) G units (**G′**)
115.0/6.74	$C_{3,5}$–$H_{3,5}$, *p*-hydroxyphenyl (**H**)
115.1/6.40-6.80	C_5–H_5, G units (**G**)
119.6/6.77	C_6–H_6, G units (**G**)

119.7/6.96	C_6–H_6, 3-dimethoxy-*para*-diphenol substructures (**G**$_{1\text{-}1'}$)
120.3/7.24	C_β–H_β, stilbene (**SB5**$_\beta$)
123.4/7.51	C_6–H_6, oxidized (H_3C–C_α=O) G units (**G′′**)
126.4/6.98	C_α–H_α, stilbene (**SB1**$_\alpha$)
126.8/7.41	C_6–H_6, oxidized (H–C_α=O) G units (**G′**)
